# Single-Channel Blind Source Separation of Spatial Aliasing Signal Based on Stacked-LSTM

**DOI:** 10.3390/s21144844

**Published:** 2021-07-16

**Authors:** Mengchen Zhao, Xiujuan Yao, Jing Wang, Yi Yan, Xiang Gao, Yanan Fan

**Affiliations:** 1 National Space Science Center, Chinese Academy of Sciences, Beijing 100190, China; zhaomengchen18@mails.ucas.edu.cn (M.Z.); wangj@nssc.ac.cn (J.W.); yanyi@nssc.ac.cn (Y.Y.); gaoxiang@nssc.ac.cn (X.G.); fanyanan@nssc.ac.cn (Y.F.); 2 University of Chinese Academy of Sciences, Beijing 100190, China

**Keywords:** spatial information network, signal reception, single-channel blind source separation, co-channel interference

## Abstract

Aiming at the problem of insufficient separation accuracy of aliased signals in space Internet satellite-ground communication scenarios, a stacked long short-term memory network (Stacked-LSTM) separation method based on deep learning is proposed. First, the coding feature representation of the mixed signal is extracted. Then, the long sequence input is divided into smaller blocks through the Stacked-LSTM network with the attention mechanism of the SE module, and the deep feature mask of the source signal is trained to obtain the Hadamard product of the mask of each source and the coding feature of the mixed signal, which is the encoding feature representation of the source signal. Finally, characteristics of the source signal is decoded by 1-D convolution to to obtain the original waveform. The negative scale-invariant source-to-noise ratio (SISNR) is used as the loss function of network training, that is, the evaluation index of single-channel blind source separation performance. The results show that in the single-channel separation of spatially aliased signals, the Stacked-LSTM method improves SISNR by 10.09∼38.17 dB compared with the two classic separation algorithms of ICA and NMF and the three deep learning separation methods of TasNet, Conv-TasNet and Wave-U-Net. The Stacked-LSTM method has better separation accuracy and noise robustness.

## 1. Introduction

In recent years, satellite networking constellations (such as onweb constellation, etc.) have developed rapidly. Satellite-to-ground communications and inter-satellite communications have further aggravated spectrum congestion, and co-frequency signal aliasing interference is inevitable [1,2,3], the signals in the electromagnetic environment of space communication show the phenomenon of time-frequency aliasing and spatial interleaving. In the real space communication, robust signal processing usually requires automatic signal separation [4,5]. In a dense constellation Internet communication, the source signal received by the receiving end may be an aliased signal of multiple channels, and due to the fact that the number of ground station observation channels is limited, the application scenario of under-determined blind source separation is extremely common. Conventional two-step method, sparse feature representation and other under-determined separation methods [6,7,8] have poor separation results under single-channel conditions and the problem of blind source separation of single-channel communication signals needs to be solved urgently. Researchers have proposed many methods to solve the single-channel separation problem, such as the ICA [9] and NMF [10] methods that use the expansion of the observation data channel to transform into the multi-channel blind source separation, but the separation accuracy needs to be improved and the generalization is poor. TasNet [11], ConvTasNet [12] and Wave-U-Net [13] based on deep learning play important role in processing the blind source separation of speech signals. The classic blind source separation method considers the physical basis [14]. However, deep learning does not. Deep learning only needs to extract useful information from big data to learn how to separate the source signal. However, these methods have their drawbacks. Large number of parameters significantly increase its computational cost and the separation accuracy performance is not good enough in Tasnet. The receiving field of one-dimensional convolution in the ConvTasNet network is smaller than the sequence length, and sequence-level modeling cannot be performed. The sampling rate of the original signal is used as the resolution in each feature map generated by convolution in Wave-U-Net, and the memory consumption is high.

This paper proposes a single-channel blind source separation method for spatial aliasing signals based on deep learning—stacked-LSTM, which is a simple and effective method to organize RNN layers in a deep structure to model extremely long sequences. The latest research on signal separation based on deep learning has proves that time-domain methods are superior to traditional time-frequency-based methods [11,12,13]. In the time-frequency method, the time-frequency representation of the mixed signal is used to obtain the time-frequency domain mask of each source signal after training. This is a commonly used signal separation method, but this method has three shortcomings [15,16,17]: Separation accuracy problems caused by the suboptimality of frequency representation, long delay problems in calculating the spectrogram, and amplitude and phase decoupling problems. First, STFT is a general-purpose signal conversion, but it is not necessarily the best for the communication signal separation task, so it will lead to low separation accuracy. Second, accurate separation requires a longer STFT time window, and this requirement increases the minimum delay of the system. Third, it is hard to accurately reconstruct the phase of the source, and an incorrect estimation of the phase will cause the accuracy of the reconstructed signal to reach the upper limit. The time domain method avoids the problems caused by directly calculating the STFT, reduces the time delay, and improves the separation accuracy. Different from the time-frequency domain method, the time-domain separation system usually receives input sequences containing a large number of time steps, which brings challenges to modeling extremely long sequences. Our proposed method solves this problem by cutting and splicing the sequences into 3-D tensors.

In the proposed method, firstly, 1-D convolution is used to extract the encoding feature representation of the mixed communication signal as input. Then, the long sequence input is divided into smaller blocks, and the Stacked-LSTM network with the attention mechanism of the SE module is trained to obtain the deep feature mask of the pure signal source. The Hadamard product of the mask of each signal source and the obtained mixed signal coding feature can be used to obtain the coding feature representation of the source signal. Finally, the 1-D convolution is used again to decode the obtained source signal characteristics to obtain the original waveform. The ratio-invariant signal-to-noise ratio is used as the training loss function to measure the accuracy of mask estimation and the accuracy of signal separation.

The main contributions of this article are as follows:

(1) A mixed communication signal data set 10-mixC is constructed using the GNURadio platform. The data set includes 10 mixed signals obtained from five types of modulation communication signals. This data set can provide data support for similar research work in the future.
(2) Stacked-LSTM method improves SISNR by 10.09–38.17 dB compared with the two classic separation algorithms of ICA and NMF and the three deep learning separation methods of TasNet, Conv-TasNet and Wave-U-Net.
(3) This method effectively improves the accuracy of blind source separation of single-channel communication signals, and has better noise robustness. It can achieve single-channel separation of 10 mixed signals such as BPSK-16QAM, 8PSK-64QAM, 8PSK-PAM4, 64QAM-PAM4, etc.

## 2. Related Work

Some traditional blind source separation methods provide solutions for single-channel blind source separation, but there are some disadvantages. Independent component analysis [9] performs well in over-determined and positive-definite blind source separation, but it performs poorly in under-determined blind source separation, especially in single-channel scenarios. Sparse Component analysis (SCA) [18] based on clustering method requires the number of mixed signals to be known. The time-frequency mask method [15] improves the separation accuracy, but there is amplitude and phase decoupling, and the short-time Fourier transform requires a higher resolution frequency decomposition window, which limits its applicability in low-delay systems. Non-negative matrix factorization (NMF) [10] can perform signal decomposition in the time domain, but it is weak in generalization ability. Single channel separation based on Kalman filter [19], LCL-FRESH filter [20] and cyclic Wiener filter [21] needs high computational complexity, and the practical effect needs to be improved.

With the development of big data and the improvement of computing power, deep learning achieves great success in time series signal processing such as speech recognition, speech separation [12,15,22,23,24,25,26,27,28,29,30,31,32,33,34,35,36,37,38], and communication signal modulation recognition [39]. These tasks demonstrate the powerful feature extraction and timing signal processing capabilities of deep learning. However, the application of deep learning in communication signal processing is mostly seen in conventional modulation recognition and classification tasks, and is involved in complex tasks such as single-channel communication signal separation. Most of the research focuses on speech signals and EGG signals. In the TasNet [11], the traditional recurrent neural network is used. Due to the difficulty of optimization, it is impossible to effectively model such a long sequence, so the separation effect is not good. ConvTasNet [12] can complete the modeling of the long sequence on the basis of the large receiving field to meet the long-term dependence of blind source separation. However, due to the limitation of the receiving field, the sequence length cannot be increased indefinitely. When the reception field of the one-dimensional convolutional neural network (1-D CNN) is small, the speech-level sequence modeling cannot be performed. Wave-U-Net [13] processes the time series context by repeatedly performing downsampling of the feature map and convolution, combining high-level and low-level features on different time scales. In each feature map generated by convolution, the sampling rate of the original signal is used as the resolution, so the memory consumption is high.

## 3. Background

### 3.1. Blind Source Separation

In the blind source separation (BSS), the waveform of the observed signal x(t) and the independence between the signal sources are used to make the estimated signal $s^{*}\left(t\right)$ as close to the signal source s(t) as possible. The source signal is expressed as $s\left(t\right)={\left\{s_{1}\left(t\right),s_{2}\left(t\right),\cdots ,s_{n}\left(t\right)\right\}}^{T}$. The received observation mixed signal is $x\left(t\right)={\left\{x_{1}\left(t\right),x_{2}\left(t\right),\cdots ,x_{m}\left(t\right)\right\}}^{T}$. The estimated signal is $s^{*}\left(t\right)={\left\{s_{1}^{*}\left(t\right),s_{2}^{*}\left(t\right),\cdots ,s_{n}^{*}\left(t\right)\right\}}^{T}$, The mathematical model of blind source separation [12,15,23,24,26,27,28,29,30,31,33,40] is linear instantaneous mixture model:

(1) x(t)=As(t)+n(t)

In the formula, A is the mixing matrix, m represents the number of source signals, and n represents the number of receiving antenna elements.When n<m, it is defined as underdetermined blind source separation. When n=1, it is defined as single-channel blind source separation under underdetermined conditions. The single-channel underdetermined blind source separation instantaneous mixing model is as follows:

(2) $$x\left(t\right)=\sum_{i=1}^{N}a_{i}s_{i}\left(t\right)$$

### 3.2. LSTM

**Figure 1:**
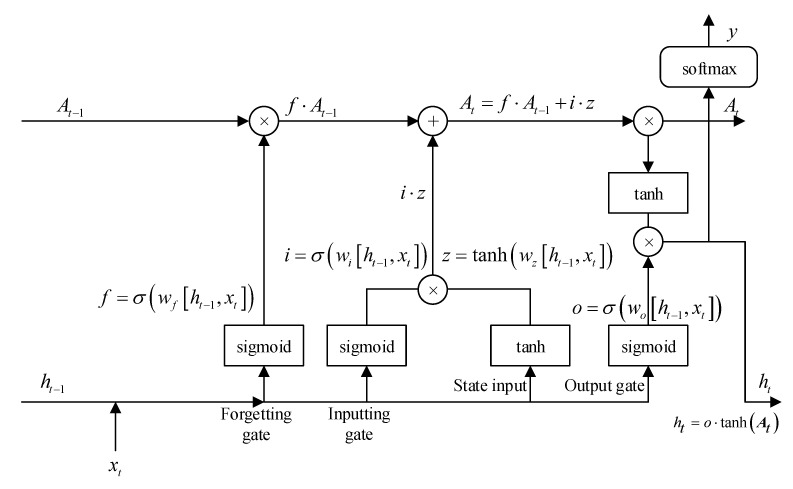
Structure of LSTM.

As is shown in Figure 1, LSTM has three control gates: input gate, output gate, and forget gate, and they jointly control the unit state A. As is shown in Formula (3), the function of the forget gate is to determine how much the state of the unit at time t−1$A_{t-1}$ is retained to $A_{t}$. $W_{f}$ and $U_{f}$ are the weights of the forget gate, and σ is the sigmoid function [41].

(3) $$f_{t}=\sigma \left(W_{f}x_{t}+U_{f}h_{t-1}\right)$$

As is shown in Formula (4), the input gate controls how much the input $x_{t}$ of the network at time t retained to $A_{t}$.

(4) $$i_{t}=\sigma \left(W_{i}x_{t}+U_{i}h_{t-1}\right)$$

As is shown in Formula (5), $z_{t}$ is used to describe the current input unit state.

(5) $$z_{t}=\tanh\left(W_{z}x_{t}+U_{z}h_{t-1}\right)$$

As is shown in Formula (6), The current unit state $A_{t}$ is calculated as follows: the last unit state $A_{t-1}$ is element-wise multiplied by the forgetting gate $f_{t}$, and then the current input unit state $z_{t}$ is element-wise multiplied by the input gate $i_{t}$, and the two products are added together. In this way, the current memory $z_{t}$ and the long-term memory $A_{t-1}$ can be combined to form a new unit state $A_{t}$ . Because of the control of the forget gate, it can save information from a long time ago, and because of the control of the input gate, it can avoid the current unimportant content from entering the memory.

(6) $$A_{t}=f_{t}\cdot A_{t-1}+i_{t}\cdot z_{t}$$

As is shown in Formula (7), the function of the output gate is to control how much the unit state at time t $A_{t}$ retains to the final output value $h_{t}$ at time t.

(7) $$o_{t}=\sigma \left(W_{o}x_{t}+U_{o}h_{t-1}\right)$$

Finally, the current unit state $A_{t}$ passes the tanh activation function, and then the result is multiplied by the output gate to get the final current moment output, as shown in Formula (8):

(8) $$h_{t}=o_{t}\cdot \tanh\left(A_{t}\right)$$

LSTM can solve the problem of gradient disappearance and explosion of RNN very well. The reason why the RNN gradient disappears and explodes is that when the number of network layers is very deep, the weight matrix W between the state and the hidden layer may be reused for the product at the same time. Suppose W has the following characteristic decomposition:

(9) $$W=Q\mathrm{diag}\left(\lambda \right)Q^{-1}$$

Then there is Formula (10):

(10) $$W_{t}={\left(Q\mathrm{diag}\left(\lambda \right)Q^{-1}\right)}^{t}=Q\mathrm{diag}{\left(\lambda \right)}^{t}Q^{-1}$$

The problem of gradient disappearance or explosion is due to the size of the eigenvalue. When the eigenvalue is greater than 1, the gradient explodes, and when it is less than 1, the gradient disappears. The above problems will lead to the inability to know the next adjustment direction of the loss function and make the learning process extremely unstable. Another problem of RNN is that when the number of network layers is too deep, the network memory function will be weakened, and there is a problem of long-term dependence. There are three solutions to the disappearance of the gradient of the recurrent neural network: first, ReLU can be selected as the activation function; second, Batch Normalization (BN) can be used, and third, the network structure can be improved, such as LSTM Network structure. LSTM can solve the problem of vanishing gradient and long-term dependence.

### 3.3. SE Module

**Figure 2:**
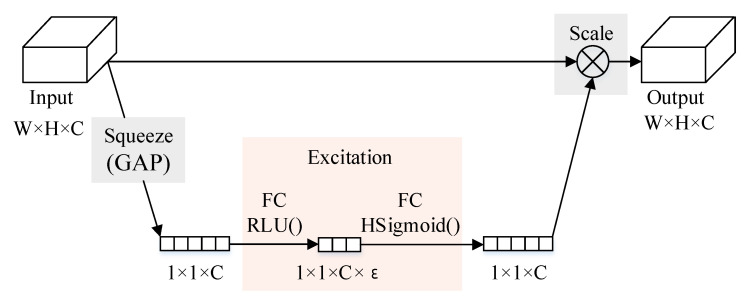
Structure of SE module.

As is shown in Figure 2, The SE (squeeze-and-excitation) module is an attention mechanism [42,43,44,45,46] that can learn to use global information to selectively emphasize useful channel information and suppress useless channel information. It learns the correlation between channels and can perform dynamic channel feature recalibration to adjust the network and improve the network expression ability. The use of the SE module is very flexible and can be added to an existing network without disturbing the original main structure of the network. The SE module mainly includes three parts: squeezing, excitation and scaling. W and H indicate the width and height of the feature map. C represents the number of channels, and the size of the input feature map is W×H×C.

The first step is the squeezing operation, which is implemented through a global average pooling. After the compression operation, the feature map is compressed into a 1×1×C vector. The next step is the excitation operation, which consists of two fully connected layers, where ε is a scaling parameter. The purpose of this parameter is to reduce the number of channels and reduce the amount of calculation. The first fully connected layer has C×ε neurons, the input is 1×1×C, and the output is 1×1×C×ε. The second fully connected layer has C neurons, the input is 1×1×C×ε, and the output is 1×1×C.

The last step is the scale operation. After the 1×1×C vector is obtained, the original feature map can be scaled. The weight of each channel calculated by the SE module is respectively multiplied with the two-dimensional matrix of the corresponding channel of the original feature map, and the result obtained is output.

## 4. Proposed Method

### 4.1. LSTM Block

**Figure 3:**
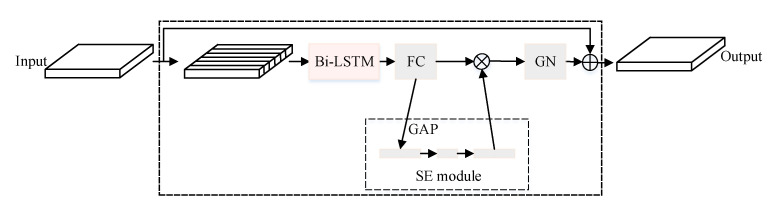
Structure of the LSTM Block.

There are three stages in the process of the sequence entering the LSTM block: the segmentation stage, the block processing stage, and the overlap and addition stage. In the segmentation stage, the sequential feature input is divided into overlapping blocks, and they are connected to form a 3-D tensor. As is shown in Figure 3, in the block processing stage, the 3-D tensor enters the Bi-LSTM sequentially in the time sequence of the segmented feature sequence, and then the fully connected layer and the SE module are connected (98). The output of the SE module is multiplied element-by-element with the output of the fully connected layer, and Group Normalization (GN) is performed on the result obtained. Then we can get the final result through residual connection. The SE module is an attention mechanism that can learn to use global information to selectively emphasize useful channel information and suppress useless channel information. It learns the correlation between channels and can perform dynamic channel feature recalibration to adjust the network and improve the network expression ability. The SE module can achieve the purpose of capturing global information with a simpler structure and a smaller network scale. Global Average Pooling (GAP) (99) is used in the SE module to regularize the entire network to avoid the risk of overfitting caused by the full connection, and it can replace the conversion function of the full connection layer. In the overlap and addition stage, the 3-D output of the last LSTM block is converted back to sequential output by performing overlap addition on the block.

### 4.2. Stacked-LSTM

**Figure 4:**
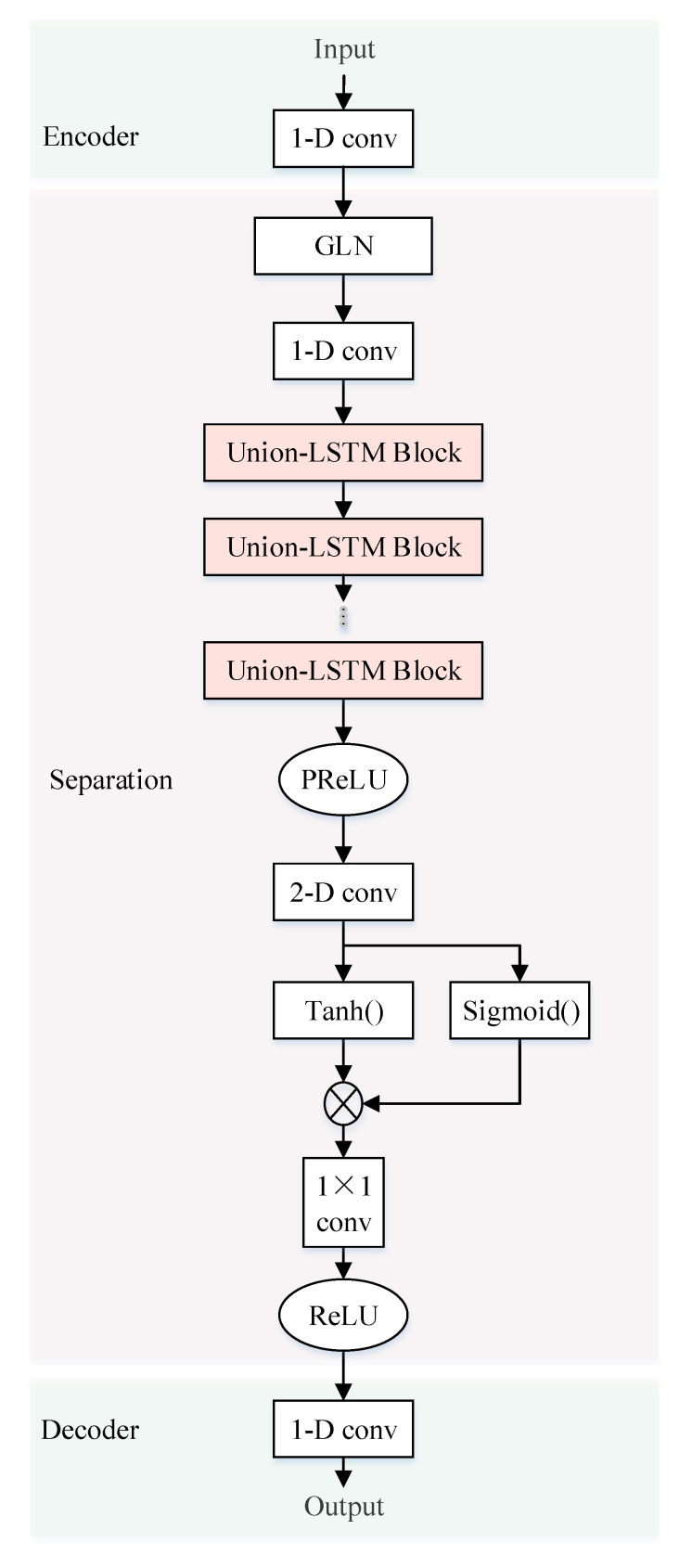
Stacked-LSTM separation network structure.

In order to effectively extract the deep features of the communication signal and improve the separation accuracy, this paper proposes a Stacked-LSTM signal separation network. The network structure of Stacked-LSTM is shown in Figure 4, which consists of three parts: an encoding part, a separation part and a decoding part. The encoding part performs feature representation on the mixed signal. The separation part is trained through the stacked LSTM block to obtain the source signal mask, and the decoding part is used to restore the waveform. Accurate separation of sequence signals requires longer time window information, that is, long-term dependence. The superior performance of LSTM in sequence signal modeling and processing can meet the long-term dependence modeling of sequence signals such as voice signals and communication signals. The structure of the LSTM unit block is shown in Figure 3.

#### 4.2.1. Linear Coding of Mixed Signals

1-D convolution is used to extract linear coding feature representation from one-dimensional aliased communication source signals. Literature [12] proves that the effect of linear coding is better than non-linear coding, so we use this coding method. The purpose of linear encoding is to encode mixed signals for subsequent processing. A total of 512 convolution kernels is used to generate the multi-dimensional coding feature of the mixed signal, and the result is used as the input of the separation network:

(11) $$x_{\mathrm{encoder}\;}=h_{\mathrm{encoder}\;}\left(x\right)=w_{1}*x+b_{1}$$

In the formula, $h_{\mathrm{encoder}\;}\left(\cdot \right)$ is the convolution operation. $w_{1}$ and $b_{1}$ are the weight and bias of the convolution kernel.

#### 4.2.2. Source Signal Mask Generation

The separation part is the process of obtaining the source signal mask. The mask of each source signal is obtained by training the network. The physical meaning of the mask actually corresponds to the mixing matrix A in the mathematical model of blind source separation. The traditional blind source separation method obtains the mixing matrix through iterative calculation, while the deep learning method obtains the multi-dimensional mapping of the mixing matrix in the neural network by training the weight coefficients. The specific steps of the separation part are as follows:

Step 1 First, perform Group Normalization (GN) (100) on the input. In the group normalization operation, the channels are grouped and the mean and variance are calculated in each group for normalization. According to the size of the batch size, the calculation of GN is independent. As an alternative to batch normalization (BN), GN can naturally transit from pre-training to fine-tuning. Its performance is better than BN, which enhances the generalization ability of the model while avoiding gradient disappearance and gradient explosion. Then the 1-D convolution is used to extract features.

Step 2 Subsequently, the feature sequence enters the stacked Union-LSTM. A stacking block contains six LSTM blocks. Then the 1-D convolution is used to further extract features.

Step 3 PReLU is used as the activation function. Since there is no zero point in the derivative of PReLU, the problem of neuron not learning in the negative interval can be prevented. Next, a 2-D convolution operation is performed to further extract features. Next, through the tanh activation function, the multi-dimensional characteristics of the mixed source signal are obtained. The result obtained by Tanh is the characteristic expression of the source signal. The result obtained by Sigmiod is the mask information of the two source signals. In this way, the time domain masks of the two source signals are obtained after training:

(12) $$s_{mask}=f_{mask}^{stacked-LSTM}\left(x_{encoder}\right)$$

In the formula, $f_{mask}^{stacked-LSTM}\left(\cdot \right)$ is separation module containing stacked-LSTM, which is used to generate a time domain mask $s_{mask}$.

Step 4 The coding feature of the mixed signal obtained in Section 4.2.1 actually contains two coding features of the source signal. Each source signal has a potential time-domain mask [18], and the coding feature of each source signal can be extracted through the time-domain mask. The obtained time-domain mask of each source signal is multiplied with the encoding feature representation of the mixed signal to obtain the feature encoding of the two communication source signals:

(13) $$s_{sep}=x_{encoder}\circ s_{mask}$$

In the formula, ∘ is the Hadamard product, which is the element-wise product of the two operands.

#### 4.2.3. Source Signal Waveform Recovery

The 1-D deconvolution is used to perform 512-dimensional feature decoding on the separated communication source signal encoding to obtain a one-dimensional time-domain waveform:

(14) $$s^{*}=h_{decoder}\left(x_{sep}\right)$$

In the formula, $h_{decoder}\left(\cdot \right)$ is the decoder.

### 4.3. Learning Process

Scale-invariant source-to-noise ratio (SISNR) [39] is usually used as the basic separation evaluation index to measure the performance of blind source separation. SISNR measures the ratio of the signal to the separation error. The higher the SISNR, the lower the separation error and the better the separation performance. Before the calculation, the source signal and the separated source signal are normalized to a zero mean value to ensure that the scale remains unchanged.

The gradient descent method is generally used during network training. This method needs to minimize the loss function. As is shown in Formula (15), a negative SISNR is used as the loss function. This ensures that in the end-to-end training, the loss is minimized and SISNR is maximized to ensure the accuracy of model training.

(15) $$\min_{\theta }\ell_{SISNR}\left(s,s^{*}\right)=-10\log_{10}\left(\frac{{∥s_{aim}∥}^{2}}{{∥s^{*}-s_{aim}∥}^{2}}\right)$$

(16) $$s_{aim}=\frac{\langle s^{*},s\rangle s}{{∥s∥}^{2}}$$

The parameters are updated through the back-propagation gradient descent algorithm:

(17) $$\theta_{encoder}\leftarrow \theta_{encoder}-\eta \frac{\partial \ell_{SLSNR}}{\partial \theta_{encoder}}$$

(18) $$\theta_{mask}\leftarrow \theta_{mask}-\eta \frac{\partial \ell_{SLSNR}}{\partial \theta_{mask}}$$

(19) $$\theta_{decoder}\leftarrow \theta_{decoder}-\eta \frac{\partial \ell_{SLSNR}}{\partial \theta_{decoder}}$$

The back-propagation gradient descent algorithm updates the parameters of the encoding part, the mask part and the decoding part $\theta =\left\{\theta_{encoder},\theta_{mask},\theta_{decoder}\right\}$. The encoder parameter set, mask parameter set, and decoder parameter set are $\theta_{encoder}=\left\{w_{1},b_{1}\right\}$, $\theta_{mask}=\left\{w_{2},b_{2}\right\}$, $\theta_{decoder}=\left\{w_{3},b_{3}\right\}$.

## 5. Experiment

### 5.1. Dataset

In the data generation and mixing part, communication data from five modulation modes, including BPSK, 8PSK, QAM16, QAM64, PAM4 were generated through the software-defined radio platform GNUradio [47], the sampling rate is 1 MHz, and the code rate is 125 K symbol/s. Referring to the existing research results [48,49,50,51,52,53] in Table 1, combined with the actual operating efficiency of the simulation platform, the selected signal-to-noise ratio range was 5–20 dB, and the step size was 2.5 dB. In the simulation, it was assumed that the aliased signals from different sources had the same frequency offset and timing deviation. This article focuses on the influence of the signal-to-noise ratio of different algorithms on the separation effect.

To meets the long-term dependence of the separation task, each type of signal generated 1000 pure data signals under each signal-to-noise ratio, and each datum contained L = 32,768 sampling points, which was 32.768 ms. First, the amplitude of the pure signal was standardized. Then we used the linear instantaneous mixing model as shown in formula (1) to mix the signals. The signals of five different modulation modes were mixed in pairs to obtain data of 10 mixing modes. In each mixing mode, the signals with the same noise ratio were mixed. The data of the 10 mixed methods were BPSK-16QAM, 8PSK-64QAM,8PSK-PAM4, 64QAM-PAM4, BPSK-8PSK, BPSK-64QAM, BPSK-PAM4, 8PSK-16QAM, 16QAM-64QAM, 16QAM-PAM4, which were used as a mixed data set, with 70,000 samples in total.

### 5.2. Experiment Process

The hardware resources used in the experiment were Tesla k80 GPU, Intel Xeon E5 and 2.60 GHz CPU, and the deep learning framework is PyTorch1.4. Five-fold cross-validation was used in all experiments.

**Figure 5:**
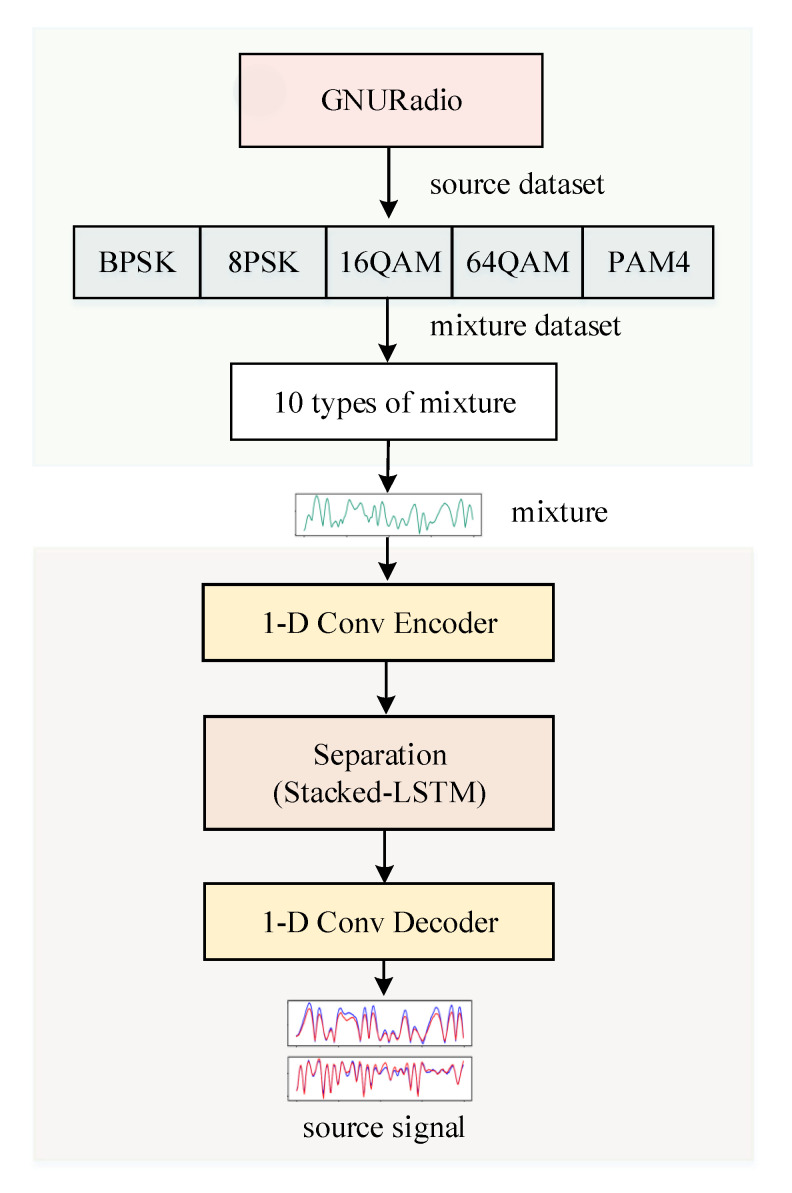
Experimental flowchart.

As is shown in Figure 5, the experimental process consists of two parts: the data generation and mixing part, and the signal separation part. In the separation part, it consisted of three modules: 1-D convolutional encoding module, separation module, 1-D convolutional decoding module.

### 5.3. Case Study 1: Effect under Pure Environment

Experimental purpose: To compare the performance difference between Stacked-LSTM network and ICA, NMF, TasNet, Wave-U-Net, and ConvTasNet under ideal transmission conditions with high signal-to-noise ratio SNR = 20 dB. The basic parameter configurations of the six blind source separation methods are shown in Table 2.

As traditional machine learning methods, ICA [9] and NMF [10] are two classic algorithms in the field of blind source separation. ICA uses dynamic embedding to convert single-channel observation data into multi-channel data for separation. It has superior performance in positive and over-determined separation, and has poor separation accuracy in single-channel under-determined separation. NMF calculates the basic matrix and coefficient matrix of the source according to the KL divergence minimization to achieve signal separation. Such traditional algorithms are equivalent to shallow models and do not extract deep features of the signal. As three deep learning separation methods, TasNet [11], ConvTasNet [12] and Wave-U-Net [13] can realize single-channel blind source separation of signals. In the TasNet network, a separation modules with three-level structure is adopted. In the separation module, Long Short-Term Memory (LSTM) network is used in each block, and its large number of parameters significantly increase its computational cost. In the 1-D block of the ConvTasNet network, a deep separable convolutional convolution is used, and the convolution kernel is an expanded convolution with different expansion rates to obtain features of different time scales. However, when the receiving field of one-dimensional convolution is smaller than the sequence length, sequence-level modeling cannot be performed. In the Wave-U-Net network, time domain convolution is used to process the time series context, combining high-level and low-level features on different time scales. The sampling rate of the original signal is used as the resolution in each feature map generated by convolution, so the memory consumption is high.

The experimental results are shown in Table 3. It can be seen that the Stacked-LSTM method had the best performance, with a loss value of −18.67 dB, which was 2.62 dB lower than that of ConvTasNet. Under ideal transmission conditions, the comparison result of separation accuracy is: Stacked-LSTM > Conv-TasNet > Wave-U-Net > TasNet > ICA > NMF algorithm, the loss was −18.67 dB < −16.05 dB < −13.97 dB < 7.93 dB < 4.09 dB. In addition, we found that the performance of the three deep learning methods under high signal-to-noise ratio was significantly better than traditional ICA and NMF, and the separation performance of Stacked-LSTM was the best method in deep learning methods.

The Stacked-LSTM network had three main characteristics, so its performance was superior. First, the LSTM block only needed to be stacked at six times, while the 1-D CNN block of ConvTasNet was stacked at eight times, and this operation was repeated three times, for a total of 24 blocks. In terms of model size, the Stacked-LSTM network was smaller. Second, the Stacked-LSTM method was based on the three-level structure of ConvTasNet, replacing the CNN block with a Bi-LSTM-based block. Multi-dimensional long sequence features were segmented and stacked into a 3-D tensor as input, which could fully model the long-term dependence of signal blind source separation, while the CNN block in Stacked-TCN had a limited receiving domain, so the Stacked-LSTM network could solve the problem of Stacked-TCN of restricted receiving domain. Third, Stacked-LSTM applied the attention mechanism of the SE module to the LSTM block. A simple mechanism was used to achieve the effect of capturing important global information.

### 5.4. Case Study 2: Effect under Noisy Environment

Experimental purpose: First, verify the generalization performance and noise robustness of the Stacked-LSTM algorithm under different noise transmission conditions. The signal-to-noise ratio range was set to SNR = 5∼20 dB. Secondly, calculate the time for the six algorithms to separate a single signal to comprehensively compare the running time and separation accuracy of the algorithm.

**Figure 6:**
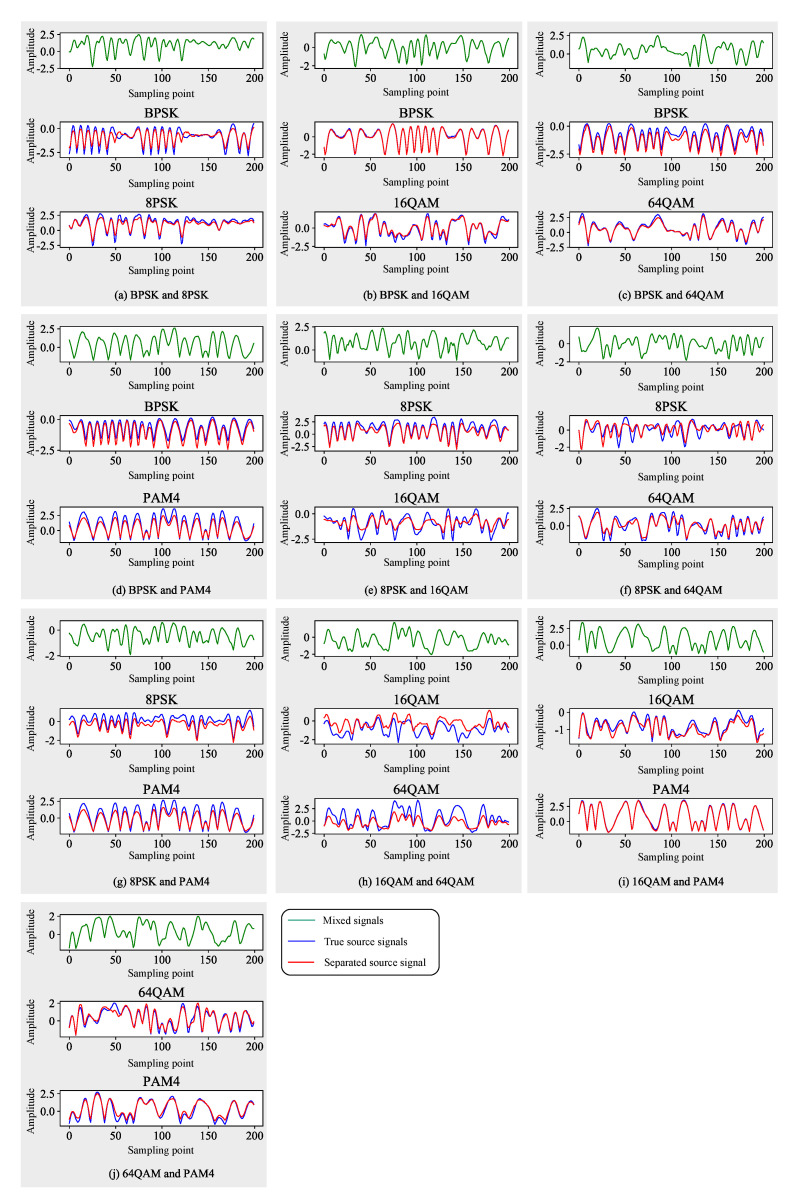
Separated signals waveform (SNR = 15 dB) (200 points).

Figure 6 is a graph of the waveform results of 10 mixed signals of the Stacked-TCN algorithm under the condition of SNR = 15 dB. It can be seen that most of the separation results were relatively good, which are basically consistent with the source signal, and have a good separation effect. Compared with the 1-D CNN block, the Bi-LSTM block could overcome the limitation of the receiving domain and convert the sequential input into a 3-D tensor, which had a higher separation potential. It was a more adequate way to model the long-term dependence of the source signal. At the same time, the attention mechanism of the SE module was added to the Bi-LSTM block, which could capture global information and obtain the correlation between different channels, thereby suppressing useless channel information, strengthening useful channel information, and dynamically adjusting the network. In addition, the excellent fitting ability of the LSTM deep neural network could better learn the time domain mask of the source signal.

**Figure 7:**
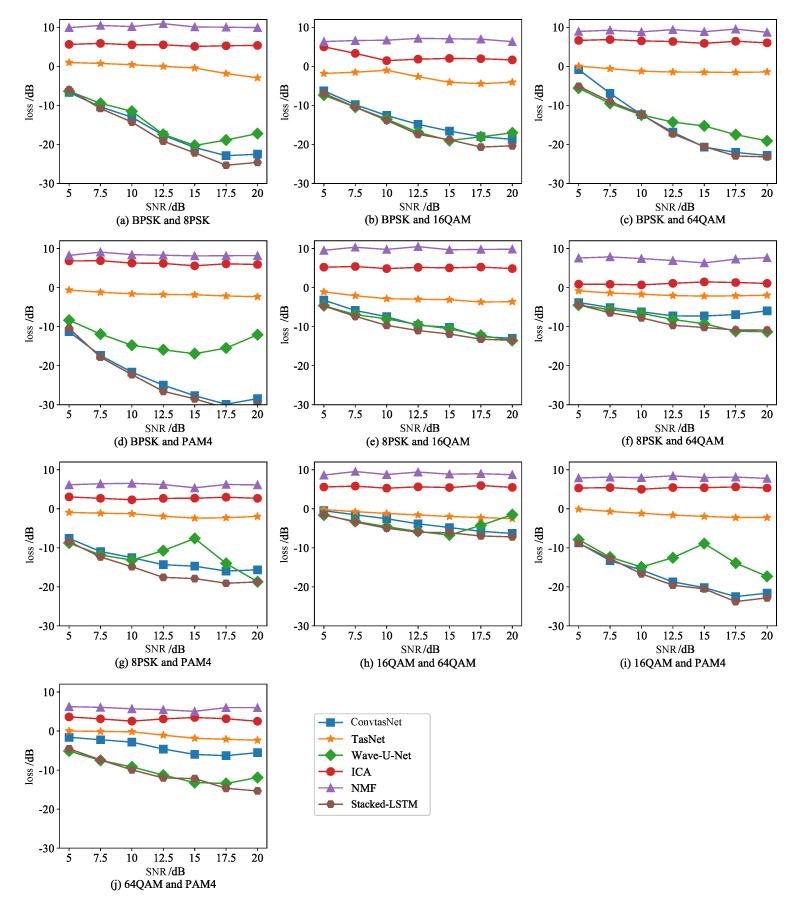
Loss under different SNR.

Figure 7 shows the loss change of the Stacked-LSTM algorithm in the range of 5–20 dB signal-to-noise ratio, and summarizes the loss line graphs of the other five comparison methods. The following experimental results were obtained: first, the three deep learning methods were significantly better than ICA and NMF machine learning methods. Second, the separation accuracy of the Stacked-LSTM method increased with the increase of SNR, and the trend was relatively stable. Third, under the low signal-to-noise ratio, the Stacked-LSTM method and the Stacked-TCN method had basically the same separation accuracy in the separation of nine kinds of mixed data. In the separation of the remaining one kind of mixed signal 64QAM-PAM4, Stacked-LSTM was superior. Compared with Stacked-TCN, Stacked-LSTM was more robust to noise interference. Fourth, from the overall 5–20 dB signal-to-noise ratio range, for the five mixed signals of BPSK-8PSK, BPSK-64QAM, BPSK-PAM4, 8PSK-16QAM, 16QAM-PAM4, Stacked-LSTM and Stacked-TCN methods were basically the same. In the other five cases, Stacked-LSTM. The method obviously had an advantage in separation accuracy.

These results are due to the fact that NMF and ICA were model-driven machine learning methods, which are essentially shallow models, suitable for tasks with small samples and precise models. However, single-channel blind source separation is a difficult problem with very little prior knowledge. The shallow model was not enough to describe its essential characteristics, and it failed to make full use of the deep characteristics and information of the signal. The ability to generalize to big data was weak. Under extreme ill-conditioned single-channel conditions, the separation performance was poor. When the task was complex and could not be accurately described by the model-driven method, the data-driven deep learning method could make up for the difficulties in the model, learn deep essential features from a large number of samples, and have strong fitting capabilities to meet the needs of tasks such as signal separation. Therefore, the separation effect of Stacked-LSTM, Wave-U-Net, TasNet, and ConvTasNet was better than that of ICA and NMF. Among them, LSTM was used as a block in the TasNet network, and a large number of parameters increased the calculation cost. In order to ensure a reasonable network model and calculation speed, the number of blocks needed to be controlled, and the modeling accuracy of the timing signal was not enough, so the effect was poor. The Wave-U-Net network processes the time series context by repeatedly performing downsampling and convolution of the feature map, which had good results, but there were fluctuations. In the ConvTasNet method, hole convolution was used in the separation module to reduce the number of parameters. The same network model size could increase the number of repetitions of the block, but the sequence receiving window was small, and sequence-level modeling could not be performed. In Stacked-LSTM, organizing LSTM in a deep structure was a simple and effective method for modeling extremely long sequences. The attention mechanism with SE module was used to capture global information, and Union-LSTM was used instead of 1D-CNN, which is separated in time domain. The mid-sequence-level modeling network had the best accuracy and stability among the three deep learning methods.

### 5.5. Separation Time Compare

In addition, the calculation time of each frame of the Stacked-LSTM time domain mask method and time-frequency domain mask method was calculated, as shown in Table 4. In most signal separation studies of time-frequency domain masking methods, the window length of STFT was at least 256 points [40,49,54,55], so the single frame duration in this experiment was 0.256 ms, and the calculation time is 1.24 ms. A longer time window and calculation time increased the minimum delay of the system. On the other hand, the Stacked-LSTM time-domain mask method could reduce the length of a single frame to 0.032 ms without reducing the separation accuracy, and its calculation time was only ms. To successfully separate the source signal from the time-frequency representation, the time-frequency domain masking method required high-resolution frequency decomposition of the mixed signal. This required a long STFT time window. This requirement increased the minimum delay of the system, which limited its applicability in real-time and low-latency applications, therefore, more and more research has begun to turn to time-domain methods [12,23,24,26,27,29].

### 5.6. Application Suggestions

The signals separated by the Stacked-LSTM network in this article are 10 mixed signals composed of five modulated signals. In fact, we can construct a training set with more modulation signals, which is expandable in the types of separated signals, and is not limited to the data set we use. The network structure and scale can be adjusted according to the amount of data, and the network can be trained to separate more kinds of signals.

## 6. Discussion

Stacked-LSTM has three advantages: first, compared with Conv-TasNet, it only requires fewer stacked LSTM blocks to achieve the same separation accuracy as the former or even surpass the former in the case of half of the mixed signals. In addition, the model is more concise. Second, it is superior to Stacked-TCN in the potential to improve separation accuracy. This is because the input of the Bi-LSTM block can be segmented and overlapped to form a 3-D tensor as input, and the longest possible sequence can be used to improve the accuracy of separation of long-term dependent effects, while the receiving domain of stacked-TCN block cannot be too large. Third, GAP is embedded in the Bi-LSTM block of Stacked-LSTM instead of the FC layer. At the same time, the attention mechanism of the SE module can be used to determine the importance of different channel features by capturing global information to dynamically adjust the network. Although Stacked-LSTM has three advantages, there is still room for improvement in separation speed.

## 7. Conclusions

In this paper, we propose a deep learning model based on Stacked-LSTM to solve the single-channel blind source separation problem, in which two source signals are mixed into one mixed signal. The proposed model can separate the mixed signals and restore them to two source signals by learning useful information from big data. Through training the time domain mask, the mapping of the hybrid matrix in the neural network is obtained, avoiding the high error of iteratively solving the hybrid matrix directly. Experiments show that the proposed model has good generalization performance not only in pure environment but also noisy environment compared with classical methods or other deep learning models. The separation loss is reduced by 10.09–38.17 dB. 

## Figures and Tables

**Table 1:** Single-channel BSS algorithms.

	Method	Source Signal	SNR (Bb)	Stride (dB)
1		BPSK	5∼30	5
2		BPSK, BPSK	5∼25	5
3		QPSK	12∼22	2
4		Radar signal	10∼30	0.5
5		EEG, ECG	/	/
6		Speech signal	−6∼9	3
7		Speech signal	/	/
8		Speech signal	/	/
9	Stacked-LSTM	BPSK, 8PSK, 16QAM, 64QAM, PAM4	5∼20	2.5

**Table 2:** Parameter configuration of five methods.

Method	Parameter Configuration	Value
ICA	Iteration number	100
NMF	Iteration threshold	$1\times 10^{-8}$
	Iteration number	100
TasNet	Base signal number *N*	128
	Frame length *L*	64
	LSTM hidden layer unit number	128
	LSTM-block number *X*	2
Wave-U-Net	Kernel size *P*	5
	Block number *X*	5
	Channel number	16-32-64-128-256
Conv-TasNet	Encoder filter number *N*	512
	Frame length *L*	16
	Bottleneck layer channel number *B*	128
	Kernel size *P*	3
	1D-block channel number *H*	512
	1D-block number *X*	8
	Repeat number *R*	3
Stacked-LSTM	The number of expected features in the input *N*	512
	The number of features in th hidden state *h*	512
	The number of hidden channels	256
	Encoder and decoder kernel size *P*	16
	Block number *X*	6
	The length of chunk *K*	200

**Table 3:** Loss of different algorithms on 20 dB mixture (dB).

Mixture	Stacked- LSTM	Conv- TasNet	TasNet	Wave- U-Net	ICA	NMF
BPSK_8PSK	−24.61	−22.51	−2.94	−17.22	5.36	9.92
BPSK_16QAM	−20.35	−18.71	−4.06	−17.02	1.64	6.32
BPSK_64QAM	−23.20	−22.83	−1.44	−19.08	6.02	8.72
BPSK_PAM4	−29.98	−28.44	−2.35	−12.09	5.92	8.19
8PSK_16QAM	−13.53	−13.00	−3.63	−13.55	4.87	9.82
8PSK_64QAM	−10.89	−5.96	−1.98	−11.31	1.07	7.67
8PSK_PAM4	−18.74	−15.64	−1.95	−18.67	2.68	6.13
16QAM_64QAM	−7.20	−6.32	−2.51	−1.51	5.48	8.73
16QAM_PAM4	−22.81	−21.61	−2.25	−17.31	5.32	7.79
64QAM_PAM4	−15.34	−5.53	−2.36	−11.92	2.50	5.98
AVG	−18.67	−16.05	−2.55	−13.97	4.09	7.93

**Table 4:** Times per frame (ms).

Method	Frame Duration	CPU Computing Time
Stacked-LSTM time domain mask method	0.032	$7.9\times 10^{-3}$
Time-frequency domain mask method	0.256	1.24

## Data Availability

Not applicable.
